# Sodium selenite alters microtubule assembly and induces apoptosis in vitro and in vivo

**DOI:** 10.1186/1756-8722-6-7

**Published:** 2013-01-17

**Authors:** Kejian Shi, Qian Jiang, Zhushi Li, Lei Shan, Feng Li, JiaJia An, Yang Yang, Caimin Xu

**Affiliations:** 1 State Key Laboratory of Medical Molecular Biology, Department of Biochemistry and Molecular Biology, Institute of Basic Medical Sciences, CAMS & PUMC, Beijing 100005, China

**Keywords:** Sodium selenite, Apoptosis, Microtubule, Cell cycle

## Abstract

**Background:**

Previous studies demonstrated that selenite induced cancer-cell apoptosis through multiple mechanisms; however, effects of selenite on microtubules in leukemic cells have not been demonstrated.

**Methods:**

The toxic effect of selenite on leukemic HL60 cells was performed with cell counting kit 8. Selenite effects on cell cycle distribution and apoptosis induction were determined by flow cytometry. The contents of cyclin B1, Mcl-1, AIF, cytochrome C, insoluble and soluble tubulins were detected with western blotting. Microtubules were visualized with indirect immunofluorescence microscopy. The interaction between CDK1 and Mcl-1 was assessed with immunoprecipitation. Decreasing Mcl-1 and cyclin B1 expression were carried out through siRNA interference. The alterations of Mcl-1 and cyclin B1 in animal model were detected with either immunohistochemical staining or western blotting. *In situ* detection of apoptotic ratio was performed with TUNEL assay.

**Results:**

Our current results showed that selenite inhibited the growth of HL60 cells and induced mitochondrial-related apoptosis. Furthermore, we found that microtubule assembly in HL60 cells was altered, those cells were arrested at G2/M phase, and Cyclin B1 was up-regulated and interacted with CDK1, which led to down-regulation of the anti-apoptotic protein Mcl-1. Finally, *in vivo* experiments confirmed the *in vitro* microtubule disruption effect and alterations in Cyclin B1 and Mcl-1 levels by selenite.

**Conclusions:**

Taken together, the results from our study indicate that microtubules are novel targets of selenite in leukemic HL60 cells.

## Introduction

Microtubules have important roles in many cell behaviors such as cell division, organelle positioning, vesicular transport and cell-shape determination [1-3]. Previous studies have showed that microtubule dynamics are necessary for these functions *in vivo*[2,4-6]. Therefore, chemicals affecting microtubule dynamics often impact these functions *in vivo*. On that basis, many anti-tumor agents have been developed for their effects on microtubule dynamics and cell-cycle distribution [7-12].

Selenium (Se) is an essential trace element [13], and appropriate selenium intake is necessary for the body to synthesize selenoproteins. Some researchers have shown that selenite concentrations that are within the nutritional range inhibit tumor formation by acting on antioxidants and in the inhibition of DNA adduct formation, the promotion of cell cycle progression and DNA repair [14-16]. However, super-nutritional levels of selenite induce endoplasmic reticulum stress, mitochondrial-related apoptosis, DNA strand breaks and cell-cycle arrest [15-19]. Therefore, many molecules, such as Akt, GADD153, P53, ERK, P38, Bad, Bim and Bax [20-24], have been reported to be involved in high-dose selenite-induced apoptosis. Additionally, super-nutritional selenite intake has been shown to be toxic to drug-resistant cancer cells and effective on tumor xenografts, which suggests that selenite has potential therapeutic effects [23-25].

In an in-depth study of selenium, selenite was reported to have strong inhibitory effects on sulfhydryl-containing proteins [15] such as tubulins, which composed microtubules [26,27], but the effects of selenite on microtubules in cancer cells had not been proven. Based on our proteomics study, proteins linked to microtubule dynamics were thought to have roles in selenite-triggered apoptosis [28]. Therefore, our study aimed to investigate the role of selenite in microtubule assembly and induction of apoptosis.

## Results

### Sodium selenite inhibits growth and induces apoptosis in HL60 cells

**Figure 1:**
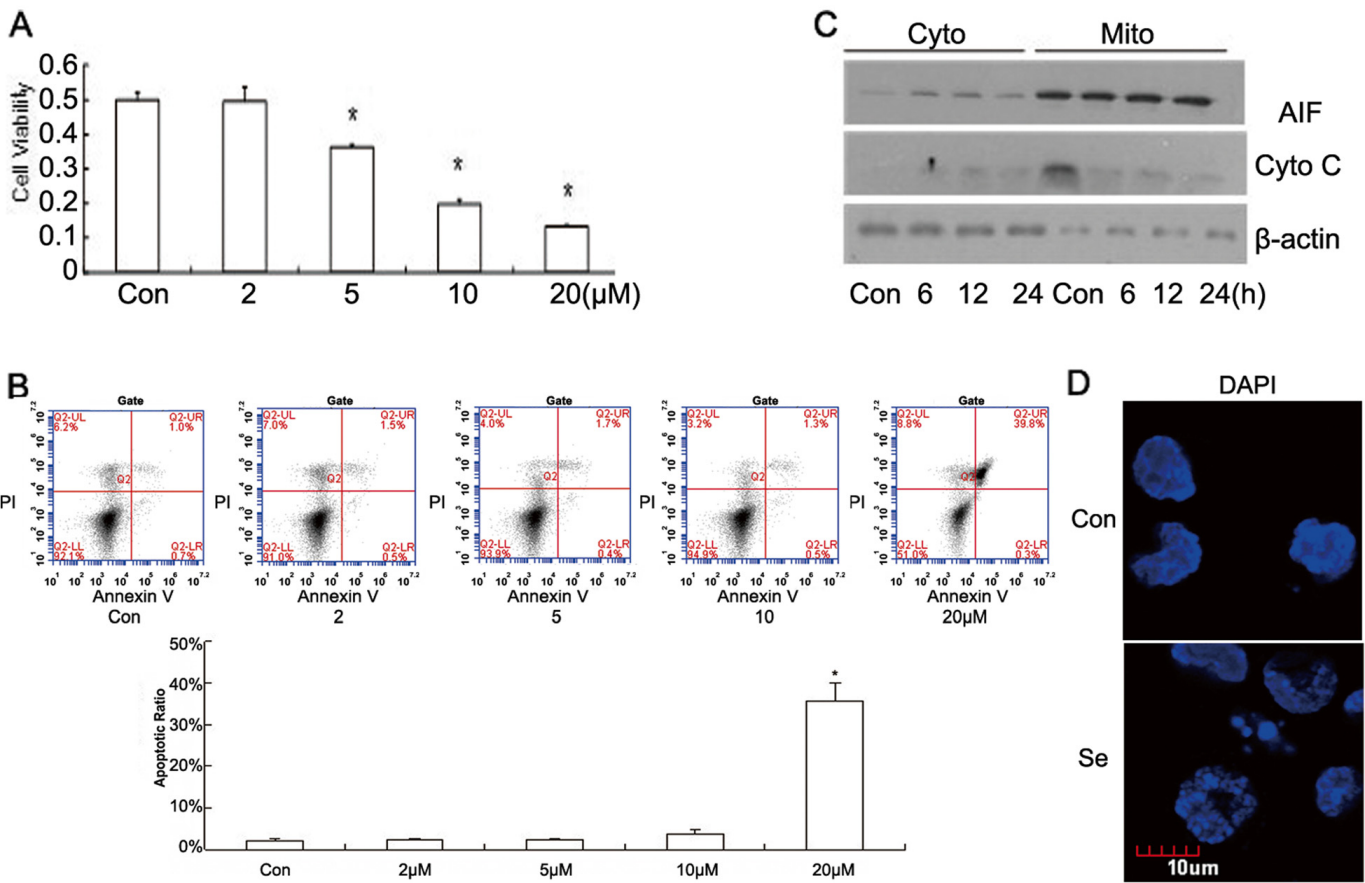
**Sodium selenite-induced, cell-proliferation inhibition and apoptosis in cultured HL60 cells. **(**A**) Sodium selenite inhibited proliferation of HL60 leukemia cells. Cells were treated with varying concentrations of selenite for 24 h, and cell viability was detected using CCK-8 kits. Mean values of three repetitions were displayed, and *P < 0.05, when compared with untreated cells. (**B**) Sodium selenite induced apoptosis in HL60 leukemia cells. Cells were collected after exposure to varying concentrations of selenite for 24 h and labeled with Annexin-V-FITC/PI, and the apoptotic ratio was detected using flow cytometry. The results were representative of three repetitions, and *P < 0.05, when compared with untreated cells. (**C**) Selenite induced the release of cytochrome C and AIF from the mitochondria to the cytoplasm. After cells were treated with 20 μM of selenite for the indicated times, fractions of mitochondria and cytoplasm were separated, and the cytochrome C and AIF contents of each fraction were detected by western blotting. This experiment was repeated at least 3 times. (**D**) Sodium selenite (20 μM) induced nucleus fragmentation of HL60 cells at 24 h, which was detected by immunofluorescence after nuclear staining with DAPI. Scale bar: 10 μm. This experiment was repeated at least 3 times.

We first observed that selenite inhibited growth of HL60 cells. After cells were exposed to differing concentrations of sodium selenite, cell viability was assessed using CCK-8 kits. Figure 1A showed that 20 μM selenite significantly reduced cell viability after 24 h. To further investigate if growth inhibition was caused by apoptosis, the cells were stained with Annexin V-FITC/PI. Flow cytometry analysis proved that typical apoptosis was elicited by 20 μM selenite (Figure 1B). To identify which pathway participated in selenite-induced apoptosis, we extracted the mitochondrial fraction of HL60 cells after they were exposed to selenite and discovered that cytochrome C and AIF were released (Figure 1C), which suggested that a mitochondrial-related apoptotic pathway was utilized. Furthermore, we observed nuclear fragmentation after staining with DAPI (Figure 1D).

### Sodium selenite alters microtubule assembly in cultured leukemia cells

**Figure 2:**
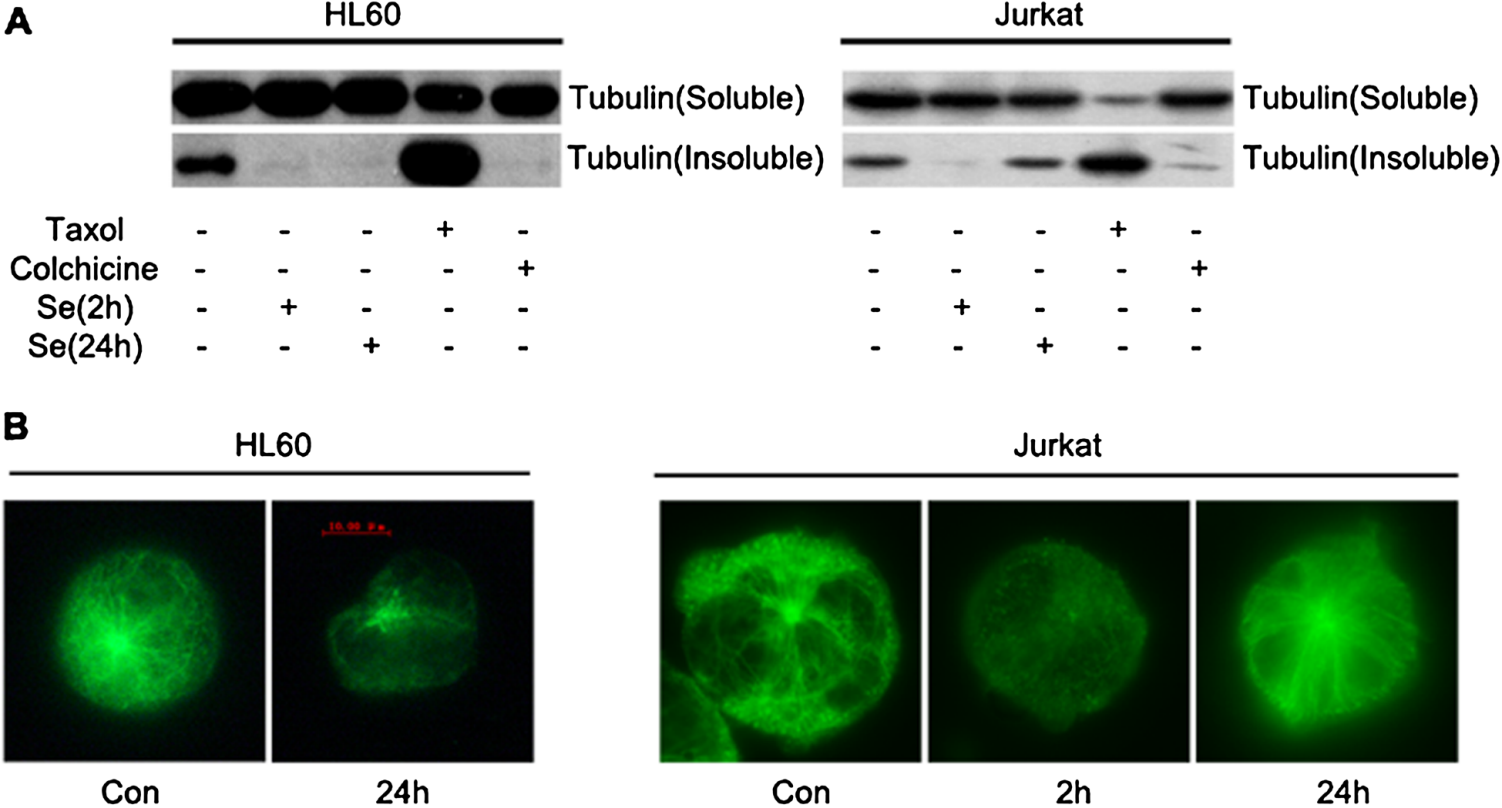
**Selenite altered microtubule assembly. **(**A**) Sodium selenite (20 μM) destroyed microtubule integrity. After exposure to 20 μM of selenite, HL60 and Jurkat cells were collected at varying times, and the insoluble and soluble fractions of tubulin were harvested and detected by western blotting. Cells that were treated with Colchicine and Taxol were used as positive controls. This experiment was repeated at least 3 times. (**B**) Microtubule depolymerization was detected by indirect immunofluorescence. The cells were harvested after selenite exposure for the indicated times, and microtubules were indirectly labeled with a β-tubulin primary antibody and FITC-conjugated secondary antibody. Scale bar: 10 μm. This experiment was repeated at least 3 times.

We then determined the mechanisms by which selenite inhibited the growth of HL60 cells. Tubulin exists in mainly two forms: soluble heterodimers that are composed of β- and α-tubulin or insoluble polymers. To detect whether selenite induced microtubule depolymerization, we extracted insoluble and soluble tubulin and discovered that 20 μM selenite destroyed microtubules. Furthermore, we observed a significant decrease of insoluble tubulin, while Jurkat cells reassembled microtubules after 2 h of selenite exposure (Figure 2A). Indirect immunofluorescence showed that microtubules presented an integrated, filamentous structure in untreated cells but were dispersive in response to selenite in HL60 cells (Figure 2B).

### Sodium selenite-treated HL60 cells are arrested at G2/M phase

**Figure 3:**
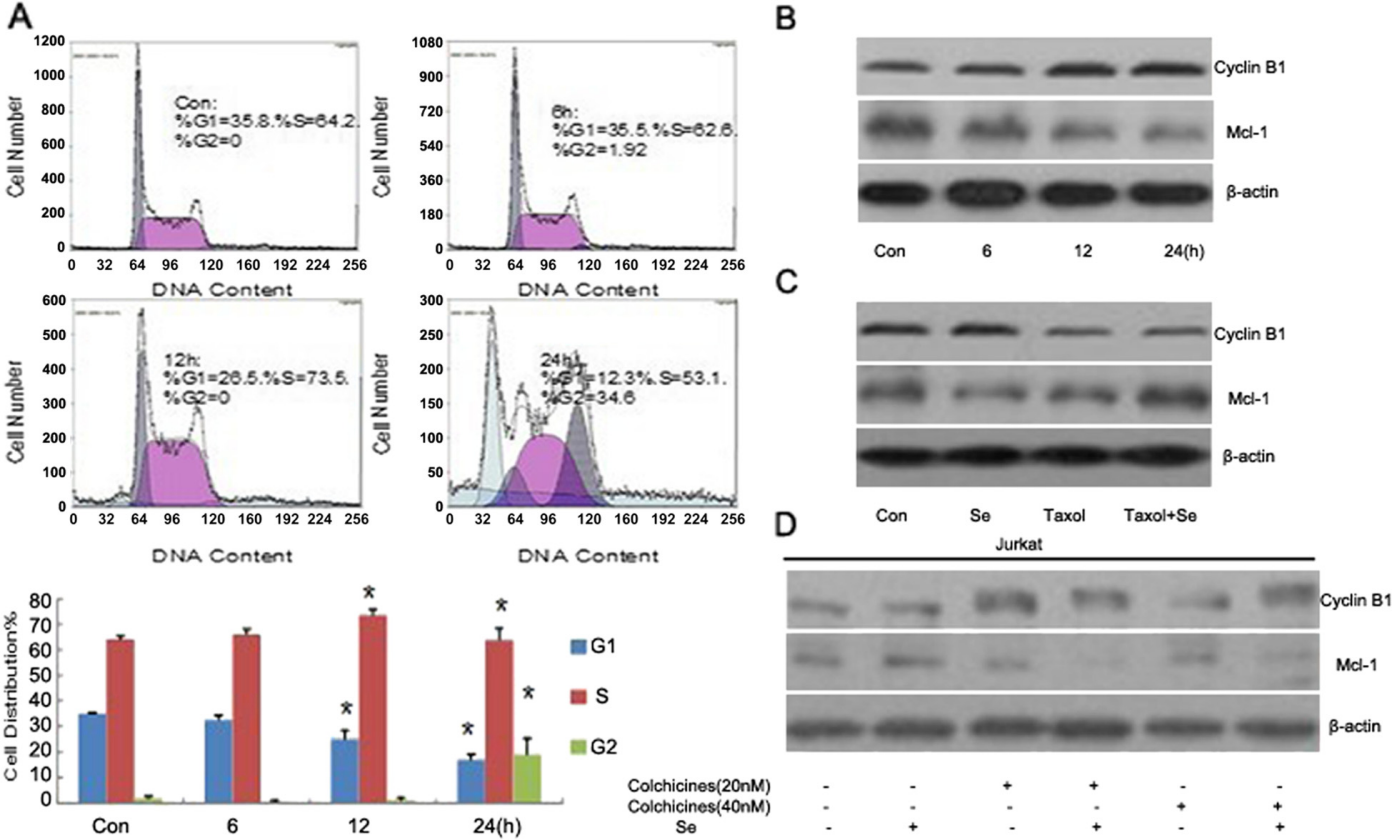
**The disruption of microtubules, which was induced by selenite, caused cell cycle arrest in HL60 cells. **(**A**) An alteration in the cell cycle distribution was detected by flow cytometry after cells were exposed to 20 μM of selenite for varying times. The experiment was repeated three times, the mean value was exhibited, and *P < 0.05, when compared to untreated cells. (**B**) Cells treated with sodium selenite (20 μM) showed increased levels of Cyclin B1 and decreased levels of Mcl-1 in a time-dependent manner. After cells were exposed to selenite (20 μM) for varying times, they were collected, and alterations in Cyclin B1 and Mcl-1 levels were detected by western blotting. This experiment was repeated at least 3 times. (**C**) Taxol reversed the change in Cyclin B1 and Mcl-1 levels. Cells were pretreated with Taxol (20 nM) for 1 h before being exposed to 20 μM sodium selenite. The cells were harvested and alterations in Cyclin B1 and Mcl-1 levels were detected by western blotting. This experiment was repeated at least 3 times. (**D**) The disruption of microtubule reorganization caused down-regulation of Mcl-1 and up-regulation of Cyclin B1 in Jurkat cells. Colchicines (20 nM or 50 nM) were added to cells after selenite exposure at 2 h. After the cells were treated with selenite (20 μM) for 24 h, they were harvested, and alterations in Cyclin B1 and Mcl-1 levels were detected by western blotting. This experiment was repeated at least 3 times.

We aimed to determine if crosstalk between microtubule disruption and the mitochondrial apoptotic pathway occurred. Because cell cycle arrest could be induced by microtubule disruption and some cell cycle-related protein kinases could regulate proteins in the mitochondria, we analyzed alterations in the cell cycle distribution of HL60 cells after selenite treatment. Compared with control, the number of cells was lower at G0/G1 phase and higher at G2/M phase after selenite treated for 24 h (Figure 3A). In addition, the protein level of Cyclin B1, a G2/M phase marker, was remarkably increased. This was concomitant with a decrease in Mcl-1, which was located in the mitochondria, in a time-dependent manner (Figure 3B). To illuminate the effect of microtubule disruption on cell cycle arrest, we employed a combined treatment of sodium selenite and Taxol (microtubule stabilizer) in HL60 cells and observed that the alteration of Cyclin B1 was abrogated (Figure 3C). As mentioned above, microtubule reorganization occurred in Jurkat cells; therefore, we destroyed microtubule reassembly through a combination of selenite and Colchicine treatments (microtubule depolymerization agent) and found that the alterations in Mcl-1 and Cyclin B1 levels were similar to those in selenite-treated HL60 cells (Figure 3D).

### Cyclin B1 interacted with CDK1 and induced down-regulation of Mcl-1

**Figure 4:**
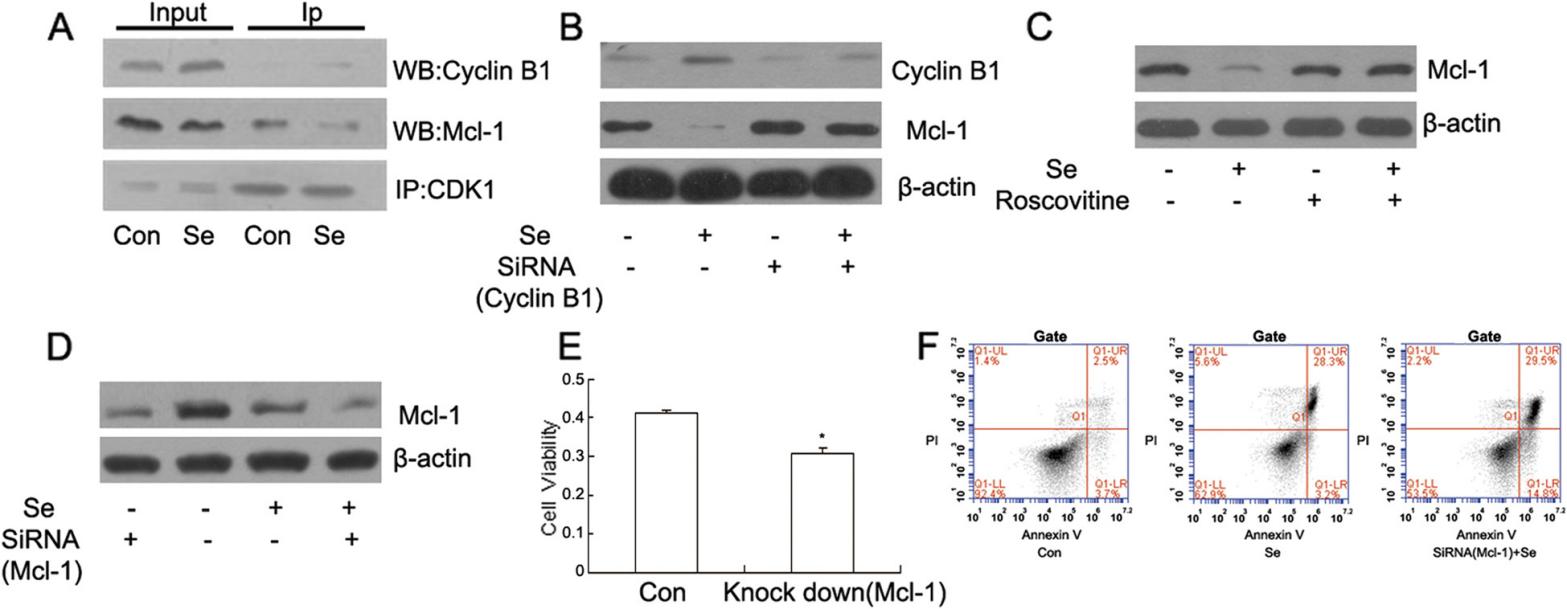
**Cyclin B1 interacted with CDK1 and induced the down-regulation of the anti-apoptotic protein Mcl-1. **(**A**) CDK1 interacted with Cyclin B1 and Mcl-1. After cells were treated with selenite for 24 h, CDK1 was immunoprecipitated from the cell lysates using a CDK1 antibody, and the CDK1, Cyclin B1 and Mcl-1 in the precipitates were detected by western blotting. This experiment was repeated at least 3 times. (**B**) Inhibiting the expression of Cyclin B1 reversed the decrease of Mcl-1. Cells were transfected with siRNAs targeting Cyclin B1 and treated with 20 μM selenite for 24 h. Cyclin B1 and Mcl-1 were detected by western blotting. This experiment was repeated at least 3 times. (**C**) Inhibition of CDK1/Cyclin B1 reversed the down-regulation of Mcl-1. Roscovitine (10μM) was added to cells for 1 h before they were exposed to sodium selenite (20 μM) for 24 h. The changes of Mcl-1 detected by western blotting. This experiment was repeated at least 3 times. (**D**) The expression of Mcl-1 was inhibited by siRNA interference. Cells were transfected with siRNAs targeting Mcl-1 and treated with selenite (20 μM). Mcl-1 levels were determined by western blotting. This experiment was repeated at least 3 times. (**E**) Mcl-1 exhibited a protective role in HL60 cells. After Mcl-1 was knocked down, cell viability was assessed using the CCK-8 kits. The experiment was repeated three times, the mean value was determined, and *P < 0.05, when compared to untreated cells. (**F**) Inhibiting the expression of Mcl-1 increased the apoptotic ratio of the cells. Cells were transfected with siRNAs targeting Mcl-1 and treated with selenite (20 μM) for 24 h. The apoptotic ratio was determined by flow cytometry after the cells were stained with Annexin-V and PI. This experiment was repeated at least 3 times.

All of the above-described observations demonstrated that microtubule depolymerization was induced by selenite and took part in the inhibition of HL60 cell cycle progression. Because Cyclin B1 is necessary in G2/M phase for the activity of CDK1, which phosphorylates and destabilizes Mcl-1, we confirmed the cellular interactions between CDK1, Cyclin B1 and Mcl-1 (Figure 4A). Furthermore, either inhibition of Cyclin B1 expression by siRNA interference or inhibition of CDK1/Cyclin B1 activity by Roscovitine rescued the down-regulation of Mcl-1 (Figure 4B,C). To further identify the protective role of Mcl-1, which was consistently altered upon cell cycle arrest, we down-regulated Mcl-1 by siRNA interference and observed HL60 cell death by cell counting using the CCK-8 kits (Figure 4D, E). Additionally, silencing Mcl-1 after selenite exposure increased the apoptotic ratio of HL60 cells (Figure 4F).

### Selenite exhibits inhibitory effects on HL60 tumor xenografts

**Figure 5:**
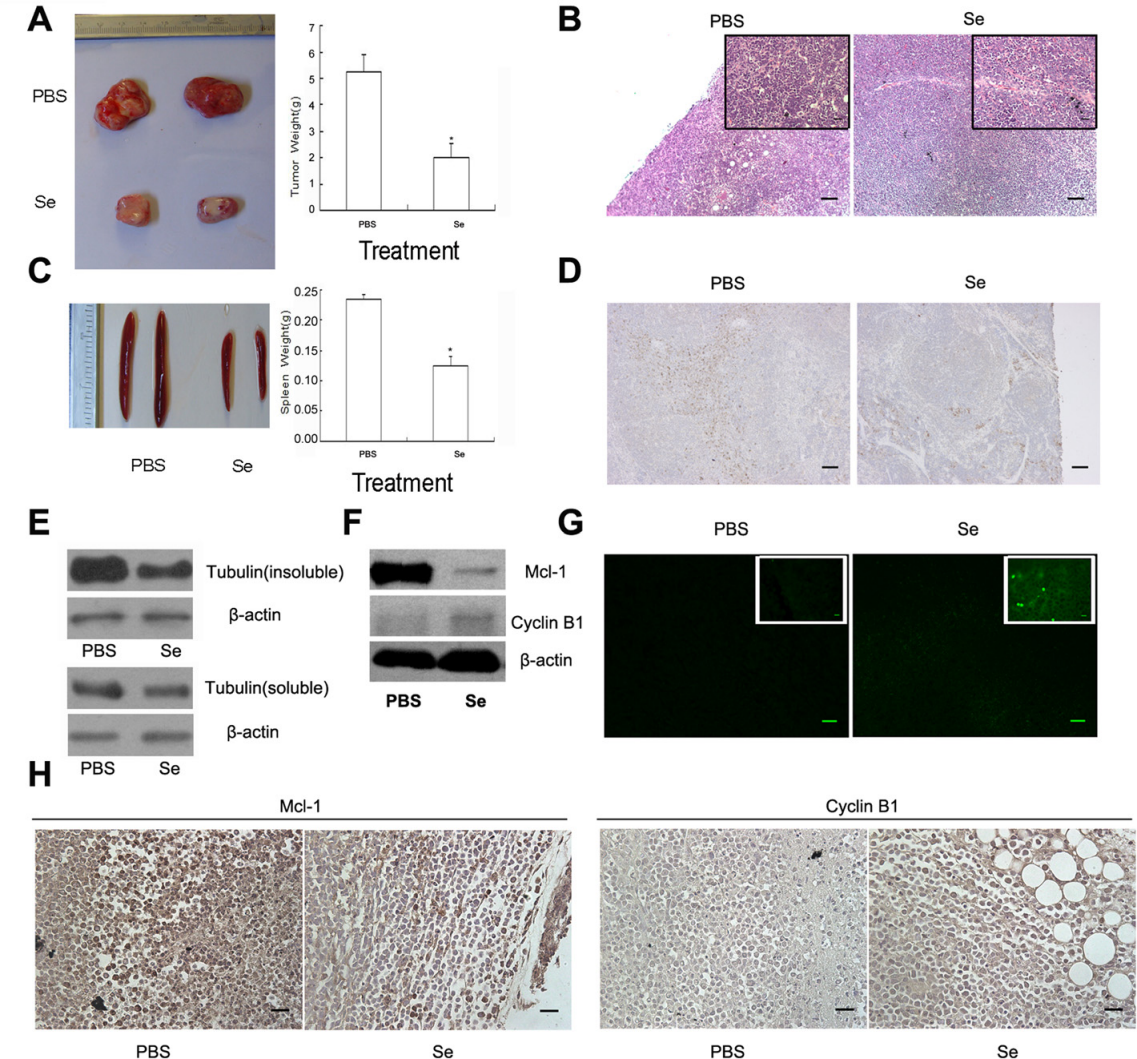
**Selenite had inhibitory effects on a HL60 xenograft tumor model. **4-week-old mice were divided into two groups randomly and each group was marked and put into its own box. The two groups lived in the same context and were fed with the same food and water. Then HL60 cells were given subcutaneously. After 1 week and tumors were detectable, PBS and selenite(1.5 mg/kg/day) were given to the control and selenite-treated group every two days respectively. After each group was injected for 3 weeks, nude mice were sacrificed for analysis. (**A**) Tumor weight decreased after selenite exposure. *P < 0.05. (**B**) Selenite caused nuclear pyknosis in tumor cells. This experiment was repeated at least 3 times. (**C**) Spleen weight decreased after selenite exposure. *P < 0.05. (**D**) Immunohistochemical staining indicated CD33 positive leukemia cells in spleen were decreased after selenite treated. Scale bar represented 100 μm. This experiment was repeated at least 3 times. (**E**) Selenite induced microtubule depolymerization in tumor cells. Soluble and insoluble-tubulin fractions were separated as indicated above and detected by western blotting. This experiment was repeated at least 3 times. (**F**) Selenite had similar effects on the regulation of Cyclin B1 and Mcl-1 as it did *in vitro. *This experiment was repeated at least 3 times. (**G**) *In situ *labeling of apoptotic tumor cells using the TUNEL assay indicated the appearance of apoptotic cells after selenite treatment. Scale bar represented 100 μm. This experiment was repeated at least 3 times. (**H**) Immunohistochemical staining indicated that alterations in Cyclin B1 and Mcl-1 levels were similar to those *in vitro. *Scale bar represented 25 μm. This experiment was repeated at least 3 times.

Finally, we determined the effects of selenite (1.5 mg/kg/day) on HL60 tumor xenografts. When compared with the control group, the volume and weight of the tumor and spleen were reduced in mice exposed to selenite (Figure 5A, C). Spleen enlargement could be caused by the infiltration of leukemic cells and it reflected the malignant degree of tumor cells. CD33 is a known marker of leukemic cells. Therefore, we detected CD33-positive cells in the spleen tissue and discovered that CD-33 positive cells decreased after selenite treatment, which also suggested that selenite had antitumor activity (Figure 5D). In addition, HE staining result indicated that selenite caused nucleus pyknosis (Figure 5B). Furthermore, *in situ* detection of apoptotic tumor cells by the TUNEL assay showed that there were more apoptotic cells after selenite treatment (Figure 5G). These results suggested that selenite had therapeutic effects on HL60 xenografts. To determine whether microtubules were depolymerized in this model, we separated soluble and insoluble tubulin and found that the amount of insoluble tubulin was decreased (Figure 5E). Furthermore, results in Figure 5F showed that selenite up-regulated Cyclin B1 and down-regulated Mcl-1 levels, which was consistent with the *in vitro* findings. Immunohistochemical staining also indicated that Cyclin B1 and Mcl-1 levels were altered similarly to those *in vitro* (Figure 5H).

## Discussion

Selenium is an essential trace element for animals, and it has been shown that super-nutritional selenite intake has anti-tumor activity [14,18,19,27,29]. Several reports have also proved the anti-tumor effects of selenite *in vivo*[24]. However, the effects of selenite on microtubule dynamics in cancer cells have not been demonstrated. Our study indicated that microtubules were a novel target of selenite.

Leynadier D et al. first discovered that selenite could directly interact with the sulfhydryl groups of β-tubulin and could inhibit microtubule polymerization *in vitro*[26]. To our knowledge, we are the first to discover that selenite also induces microtubule depolymerization in HL60 cells and *in vivo*. However, because microtubules reorganized in Jurkat but not in HL60 cells, the apoptotic mechanisms of the two cell lines differed. We mainly investigated the mechanisms by which selenite induced apoptosis. Because tumor cells have a strong ability to replicate themselves and tubulins, which compose spindles, are essential for this process, we speculate that selenite-induced apoptosis is at least partly dependent on the effects of selenite on microtubules. Therefore, the growth inhibitory effect of selenite on cultured HL60 cells was assessed, and we discovered that 20 μM of sodium selenite significantly inhibited cell growth. Furthermore, Annexin V-FITC/PI double staining assay proved that selenite-induced apoptosis occurred and nuclear fragmentation was witnessed in selenite-treated cells. Last, we discovered that cytochrome C and AIF were released from the mitochondria to the cytoplasm, which suggested that selenite-induced apoptosis in HL60 cells might be associated with the mitochondrial apoptotic pathway. Cell cycle-related proteins that were consistently altered with microtubule dynamics could regulate Bcl-2 family members, which were located in the mitochondria. Therefore, we speculated that selenite inhibited HL60 cell growth through its effects on microtubules.

Several reports suggested that microtubule-interfering drugs affected cell cycle distribution by regulating the activity of CDKs and, therefore, altering protein phosphorylation at different cell cycle phases [11]. Mcl-1, a Bcl-2 family member, is regulated by the Cyclin B1/CDK1 complex and is linked to the mitochondrial apoptotic pathway by binding and inhibiting pro-apoptotic proteins [12,30,31]. Our current study proved that selenite could induce cell cycle arrest and remarkable alterations of Cyclin B1 and Mcl-1 levels in HL60 cells through its effect on microtubule depolymerization. Interestingly, a combination treatment of Colchicine and selenite in Jurkat cells up-regulated Cyclin B1 and down-regulated Mcl-1. The observations in Jurkat cells also supported the relationship between microtubule destruction and alterations in Cyclin B1 and Mcl-1 after selenite exposure. Cyclin B1 is necessary for the activity of CDK1, which phosphorylates and destabilizes Mcl-1 [32-36]. We observed that Cyclin B1 interacted with CDK1. Furthermore, either siRNA interference of Cyclin B1 or inhibition of the CDK1/Cyclin B1 complex with Roscovitine rescued the decrease of Mcl-1. Further investigation confirmed the protective role of Mcl-1 on HL60 cells and suggested that the growth inhibitory effects of selenite might be associated with the down-regulation of Mcl-1. Finally, a combination of siRNA targeting Mcl-1 and selenite treatment caused a higher apoptotic ratio than selenite treatment alone. These results supported our conclusion that selenite altered microtubule assembly and inhibited HL60 cell growth through cell cycle arrest and decrease in Mcl-1 levels.

The above-described experiments indicated that selenite altered microtubule assembly and induced cell cycle arrest in HL60 cells. To identify the therapeutic activity of selenite *in vivo*, we established a HL60-cell-bearing nude mice model. Experiments *in vivo* showed that selenite inhibited tumor growth and induced nucleus pyknosis. Furthermore, we also found that selenite depolymerized microtubules *in vivo*. Additional experiments demonstrated that alterations of Cyclin B1 and Mcl-1 levels in the nude mice model were similar to those findings *in vitro*, which suggested that the mechanisms demonstrated *in vitro* were also active at the tissue level.

## Conclusions

In conclusion, the microtubule destruction that was induced by selenite stimulated the apoptotic pathway by up-regulating Cyclin B1, which interacted with CDK1 and destabilized the anti-apoptotic protein Mcl-1. We also found that sodium selenite had therapeutic functions in a HL60-cell-bearing nude mice model through its microtubule destruction effects. Importantly, this investigation explored the effects of selenite on apoptosis in a distinct way.

## Materials and methods

### Chemicals and antibodies

Roscovitin, anti-β-Tubulin (2-28-33) and anti-β-Actin (AC-15) antibodies were obtained from Sigma-Aldrich. Anti-Cyclin B1 and anti-Mcl-1 antibodies, which were used for western blotting, were obtained from Cell Signaling Technology. For immunohistochemical staining, an anti-Cyclin B1 antibody was purchased from Excell, an-anti CD33 antibody was purchased from BIOSS and an anti-Mcl-1 antibody was purchased from Santa Cruz. The Cdc2 (1/Cdk1/Cdc2) antibody was purchased from BD Biosciences Pharmingen. HRP-conjugated anti-mouse and anti-rabbit antibodies were purchased from ZSGB-BIO. A FITC-conjugated anti-mouse antibody was purchased from Jackson.

### Cell culture

HL60 and Jurkat cells were grown in RPMI 1640 medium containing 10% advanced fetal bovine serum, 100 units/mL penicillin and 100 units/mL streptomycin and incubated in a humidified, 5% CO_2_ incubator that was set at 37°C.

### Indirect immunofluorescence microscopy

HL60 cells (8 × 10^5^ total) were harvested. The cells were transferred to slides, fixed in 4% paraformaldehyde and permeabilized using 0.1% Triton X-100. After the slides were blocked with 2% BSA, the cells were incubated with β-tubulin antibody overnight at 4°C. After washing with PBS three times, the cells were incubated with FITC-conjugated secondary antibody for 60 min at room temperature. After a second round of washing, the cells were stained with DAPI for approximately 5 min, and the slides were washed three times and mounted in anti-fading medium. Images were visualized using a Zeiss microscope (Carl Zeiss, Jena, Germany).

### Western blotting

Approximately 1 × 10^6^ cells were collected for each treatment. After washing with ice-cold PBS, the cells were resuspended in RIPA lysis buffer (20 nM Tris, pH 7.5; 1 mM EDTA; 1 mM EGTA; 150 mM NaCl; 1% Triton X-100; 2.5 mM sodium pyrophosphate; 1 mM β-glycerolphosphate; 1 mM Na_3_VO_4_; 1 mM PMSF; and 1 μg/mL leupeptin) and were submitted to ultrasonication on ice. The lysates were centrifuged at 12,000 × *g* for 20 min at 4°C, and equal amounts of proteins were separated by SDS-PAGE. The proteins on the PAGE gel were then transferred to a nitrocellulose membrane. After being blocked with 5% non-fat milk, the membranes were washed with TBST and incubated overnight with primary antibody at 4°C. After being washed three times with TBST, the membranes were incubated with a HRP-conjugated secondary antibody for approximately 1 h at room temperature. Subsequently, the membranes were washed another three times and probed with supersignal chemiluminescent substrate.

### Immunoprecipitation

Cells (1 × 10^7^) were harvested and washed twice with ice-cold PBS. The pellets were resuspended in RIPA buffer and lysed on ice for 30 min. Subsequently, the lysates were centrifuged at 12,000 × *g* for 20 min at 4°C. A suitable amount of cdc2 antibody was added to the protein lysate (200 μg) and rotated overnight at 4°C, while the remaining protein was used as input. Protein A + G was added, and the mixture was rotated for another 3 h at 4°C; then, the samples were washed with RIPA buffer three times. Finally, the beads were resuspended in 3 × SDS loading buffer and boiled for 10 min. After a short centrifugation step, the supernatant was collected.

### siRNA interference

siRNAs targeting Cyclin B1 (5^′^-CCAAACCTTTGTAGTGAAT-3^′^), Mcl-1 (5^′^-GGACTGGCTAGTTAAACAA-3^′^) and negative controls for each sequence were synthesized by GenePharma. Approximately 1 × 10^7^ cells were collected and washed with Opti-MEM medium (Gibco). Then, the cells were transfected with 200 nM siRNA and RNAiMAX in Opti-MEM. After transfection for approximately 12 h, the cells were treated with sodium selenite for 24 h.

### Detection of cell cycle distribution

Approximately 1 × 10^6^ cells were collected and fixed in 70% ethanol overnight at 4°C. Each sample was centrifuged at 1,000 × *g* for 10 min at room temperature and washed with ice-cold PBS. Subsequently, the cells were incubated with 50 μg/ml RNase in PBS for 30 min at 37°C. After adding PI to the cells at a final concentration of 50 μg/mL, we detected the absorption at 620 nm by flow cytometry.

### Detection of apoptosis with AnnexinV-FITC/PI staining

The cells (1 × 10^6^) were harvested and washed twice with ice-cold PBS. Subsequently, the cells were stained with AnnexinV-FITC in binding buffer in the dark for 15 min. After being centrifuged at 1,000 × *g* for 10 min, the cells were resuspended in binding buffer containing PI. Finally, the apoptotic ratio was determined by an Accuri C6 flow cytometry. We calculated the apoptotic ratio by calculating the sum of Annexin V^+^/PI^-^ cells’ ratio and Annexin V^+^/PI^+^ cells’ ratio.

### The effect of sodium selenite on the viability of HL60 cells

HL60 cells were seeded into a 96-well plate at a concentration of 40,000 cells per well. After treatment with varying concentrations of sodium selenite for 24 h, cell viability was assessed using CCK-8 kits (Dojindo Molecular Technologies, Tokyo, Japan).

### The *in vivo* microtubule polymerization assay

An established method was modified and used to separate insoluble tubulin from soluble tubulin [30]. Approximately 2 × 10^6^ cells were collected and washed twice. The cells were then resuspended in hypotonic buffer at 37°C for 5 min. After centrifugation at 14,000 × *g* for 10 min at 25°C, the supernatants, which contained soluble tubulin, were collected, and the pellets containing insoluble tubulin were resuspended in RIPA buffer and subjected to ultrasonication on ice. The lysates were centrifuged at 12,000 × *g* for 10 min at 4°C, and the supernatants were collected.

### Xenograft tumor model

At the beginning of the experiment, 4-week-old female mice were chosen and divided into control or selenite-treated group randomly. Each group was marked and put into its own box. The two groups lived in the same context and were fed with the same food and water. Leukemia HL60 cells were inoculated subcutaneously into female nude mice. After tumors were palpable, an intraperitoneal injection of sodium selenite dissolved in PBS was given to each mouse every 2 days (1.5 mg/kg/day) for 3 weeks, and the control group was treated with PBS for the same period time. At the end of the experiment, the mice were sacrificed, and the tumors and spleens were rapidly removed and weighed. The Declaration of Helsinki and the guide for Laboratory Animal Care and Use were maintained.

### Immunohistochemical staining

The slides were deparaffinized in xylene and dehydrated with decreasing concentrations of ethanol. After the slides were washed with running water for 2 min, endogenous peroxidase was blocked with 3% peroxide that was dissolved in methanol. Subsequently, the slides were immersed in boiled sodium citrate for antigen retrieval, and after being washed three times with 0.01 M PBS for 5 min, the slides were incubated with either anti-Mcl-1 or anti-Cyclin B1 antibody overnight at 4°C. The slides were incubated with secondary antibody at room temperature for 3 h, treated with DAB, stained with Mayer’s hematoxylin for 2 min and washed with running water. Slides were then dehydrated with increasing concentrations of ethanol and clarified with xylene. Finally, the slides were mounted with medium.

### HE staining

After dehydration with decreasing concentrations of ethanol as described above, the slides were stained with Harriss hematoxylin for 15 min and washed for 3 min. Then, the slides were immersed in 1% hydrochloric acid dissolved in 75% ethanol for 30 s. Before dehydration, the slides were stained with eosin for 10 min. Finally, the slides were clarified with xylene and mounted with medium.

### TUNEL assay

A FragEL™ DNA Fragmentation Detection Kit was purchased from MERCK. The slides were deparaffinized in xylene and dehydrated with decreasing concentrations of ethanol. After washing with 1 × TBS for 2 min, the slides were incubated with 20 μg/ml proteinase K for 20 min at room temperature. The slides were then washed with 1 × TBS and incubated with 1 × TdT buffer for approximately 30 min at room temperature. Subsequently, the slides were incubated with 57 μl of mix buffer and 3 μl of TdT enzyme for 60 min at 37°C. After being washed three times with 1 × TBS, the slides were mounted with medium.

### Statistical analysis

The values were represented as mean ± SEM. Two-tailed students’ t-tests were used for two groups comparison analysis, and P < 0.05 was considered to be significant. The bar charts were used to reflect the alterations of experimental data [18,24,37,38].

## Competing interests

The authors declare that they have no competing interests.

## Authors’ contributions

KS designed and finished most of the project, and drafted the manuscript. QJ and ZL helped KS design the project. QJ, LS and FL helped KS establish the HL60 cells bearing nude mice model. Indirect immunofluorescence staining was performed by JA. Statistical analysis was performed by YY. CX is the supervisor of all authors and provides some helps in manuscript editing and project design. All authors read and approved the final manuscript.

## Authors’ information

First author: Kejian Shi.
